# TLR7/8 signaling balances cytokine responses in neonatal monocytes

**DOI:** 10.1038/s41598-026-46534-6

**Published:** 2026-04-13

**Authors:** S. Dreschers, E. Heiler, L. Oppermann, C. Gille, M. Hornef, T. W. Orlikowsky

**Affiliations:** 1 Department of Neonatology, University Children’s Hospital Aachen, Aachen, Germany; 2 Department of Neonatology, University Children’s Hospital Heidelberg, Heidelberg, Germany; 3 Institute of Medical Microbiology, University Hospital Aachen, Aachen, Germany

**Keywords:** Monocytes, Neonatal TLR signaling, NFκB signaling, Neonatal sepsis, Sustained inflammation, Immunology, Microbiology

## Abstract

Newborns are particularly vulnerable to nosocomial infection, but the underlying mechanisms are incompletely understood. Age-dependent differences in the expression of innate immune receptors and thus a reduced or delayed antimicrobial host response might contribute. Here we discovered that neonatal cord blood monocytes (CBMo) express lower levels of Toll-like receptor TLR8 but similar levels of TLR7 as compared to adult peripheral blood monocytes (PBMo). We investigated the TLR7/8 signal transduction by stimulating monocytes of adults (PBMo) and neonates (CBMo) with imiquimod (R_837_) and resiquimod (R_848_). Toll-like receptors (TLRs) bind various constituents of invading pathogens and trigger defense reactions. We found differential expressions of TLR7 and TLR8 which both bind to ssRNAs. We could show, that inflammatory cytokine response represented by TNF-α is comparable in PBMo and CBMo. This effect was stronger in resiiquimod (R_848_) treated PBMo and CBMo. Whereas IL-6 secretion was found similar induced in PBMo and CBMo after stimulation with TLR7/8 ligands, IL-10 was only induced in PBMo, biasing the cytokine answer to the pro-inflammatory response in CBMo. Cytokine response was more dependent to NFκB signaling although MAP kinase activation was required for the IL-6 response in CBMo. The type 1 interferon response was found comparable in PBMo and CBMo and was correlated to IRF-7 activation. We utilized an in vitro model of gram-negative bacterial (*E. coli*) infection to study whether TLR7/8 pre-stimulation influence peri-phagocytic reactions. TLR7/8 pre-stimulation modified the *E. coli* PAMP receptor CD14 and CD32 expression. IL-6 and IL-10 secretion was not altered by TLR7/8 stimulation prior to infection. TLR7 pre-stimulation reduced *E. coli* survival in CBMo, but was not accompanied by higher TNF-α levels. Administration of imiquimod (R_837_) displays therefore beneficial aspects for opportunistic bacterial infections *in-vitro*

**Supplementary Information:**

The online version contains supplementary material available at 10.1038/s41598-026-46534-6.

## Introduction

Activation of the innate immune system is mediated by stimulation of pattern recognition receptors (PRR) such as Toll-like-receptors (TLR), NOD-like receptors, and helicases such as RIG-I. The antimicrobial host response protects the host against pathogen invasion and tissue spread as well as organ damage. TLRs sense a broad spectrum of bacterial and viral molecules. TLR3 binds preferentially viral double-stranded RNA (^44^ Kulka et al), whereas TLR7 and TLR8 exhibit a high affinity to single-stranded RNA^1,2^. In contrast, TLR9 is a sensor for unmethylated double stranded DNA found for example in bacteria^3^ but also after mitochondrial damage and in herpes virus-infected cells^4^. Interestingly, the nucleic acids sensing TLRs such as TLR3, TLR7/8 and TLR9 are located in an endosomal compartment^5^, whereas the TLRs recognizing structures like bacterial cell wall constituents (lipopolysaccharide or diacyl- and triacyl-lipopeptides) or flagellin (TLR1, -2, -4, -5, and -6), are mainly located at the host cell plasma membrane. Infections with bacterial pathogens activate a panel of TLRs and will first encounter TLR1, -2, -4, -5, and -6, but intermediates of bacterial replication and transcription may also be detected by oligonucleotide sensing TLRs mentioned above^6^.

Both TLR7 and 8 are initiating a complex signal cascade. A common feature is recruitment of the adaptor protein MYD88, a prerequisite to transfer the signal to downstream factors^7^. TLR7 then via IRAK4 activates MAPKs, which induce the expression of IL-1ß, IL-6 and IL-23. TLR8 via IRAK4 activates IKK and NFκB stimulating expression of IL-6, IL-12, IL-27 and TNF-α. Alternatively, both TLR7 and 8 can engage TBK1 which activates signaling via IRF family proteins leading to expression of type I interferons (IFN-α and -β)^6^. The activation of TLR7 and TLR8 signaling is cell type-dependent and tightly regulated. Plasmacytoid dendritic cells (pDCs) and B-cells express TLR7, whereas myeloid cells such as monocytes (Mo) and macrophages (MΦ) strongly express TLR8. Recent studies indicated that the transport to the appropriate cellular compartment, the degradation via ubiquitinylation and glycosylation is controlling TLR function^8^. Also, TLR stimulation may alter the expression of other TLRs and thereby alter the response to an ongoing or subsequent pathogen exposure.

Newborns are more vulnerable to infection with both viruses and bacteria and may even suffer from co-infections. For example, newborns suffering from bronchopulmonary disease (BPD) are susceptible to RSV infection^9^ and prospective studies reported that 20–50% of hospitalized RSV-infected children were co-infected with bacteria^10^. Superinfection was associated with an overall more severe disease, prolonged hospitalization, and the requirement for treatment in an ICU^11^. Also, the incidence of necrotizing enterocolitis (NEC), a strongly proinflammatory condition driven particularly in preterm infants by the enteric microbiota, was increased during the SARS-CoV2 pandemic^12^. On the molecular level, the induction of TLR7/8 signaling by a primary virus infection may be beneficial or detrimental for the host in regard to a subsequent secondary bacterial infection.

Therefore, we here analyzed in an in vitro stimulation assay of primary adult blood monocytes and cord blood monocytes, how a primary viral challenge may modulate the immune response of the host to a secondary bacterial challenge in the neonatal host. We substituted viral exposure by stimulation of TLR7 and TLR8 with the imidazoquinoline compounds imiquimod (R_837_) and resiquimod (R_848_), and the secondary bacterial challenge by co-incubation with the gram-negative bacterium *E. coli*. TLR7 and TLR8 expression was assessed on the protein level and potential synergistic effects on phagocytosis and cytokine expression were monitored.

## Results

### TLR7/8 expression in PBMo and CBMo

**Fig. 1:**
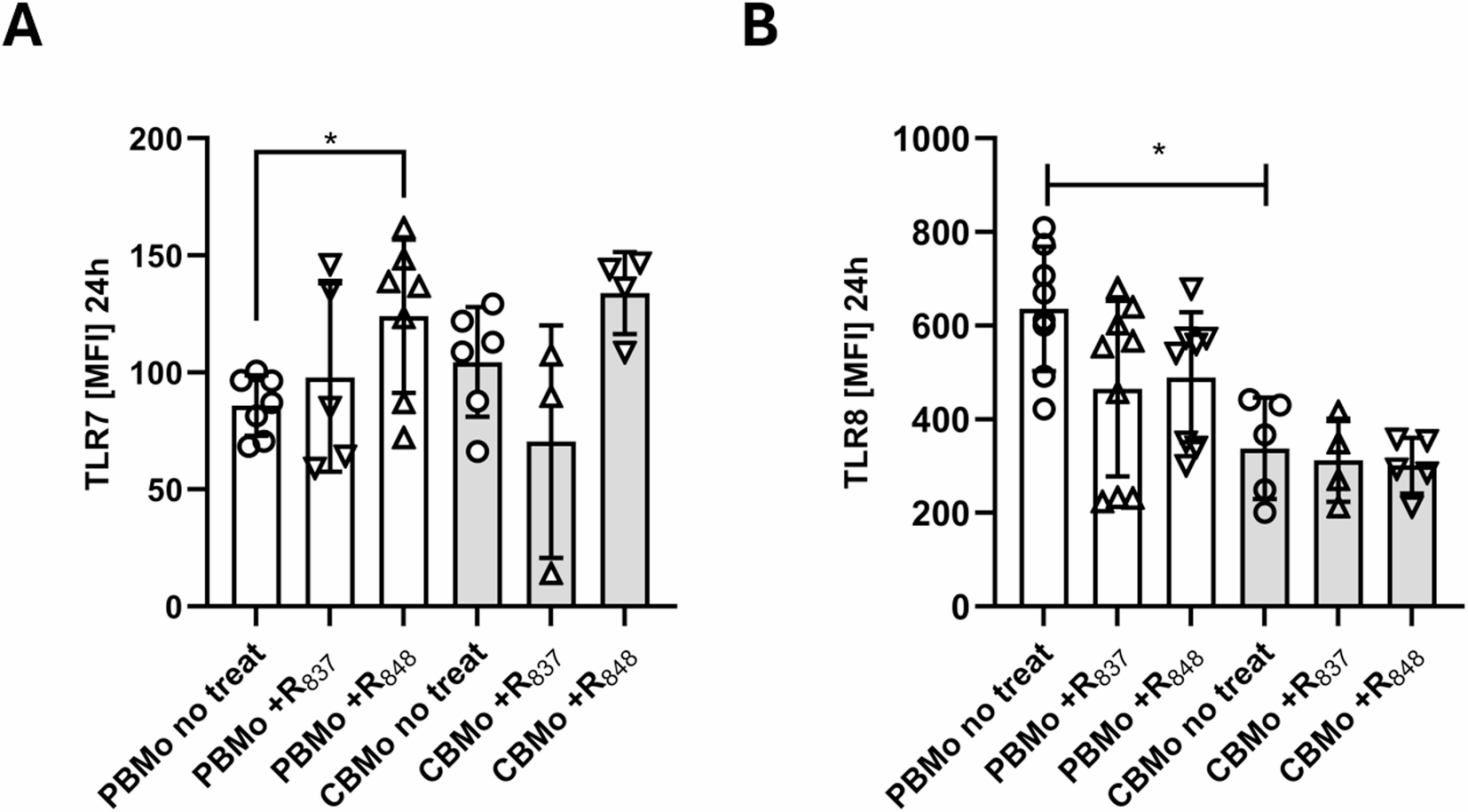
TLR7/8 expression density by PBMc and CBMc without or with imiquimod (R_837_) or resiquimod (R848) stimulation*.* TLR7 (**A**) and TLR8 (**B**) expression density in PBMo (white) and CBMo (grey) that were treated as indicated. Imiquimod (R_837_) or resiquimod (R_848_) for 24 h (* *p* < 0.05 *p* represents non-paired Student’s *t*-test; blunt-ended bars, two-way ANOVA, * *p* < 0.05).

First, we analyzed the expression level of TLR7 and TLR8 in PBMo and CBMo by flow cytometry (Fig. 1A, B). In unstimulated cells, TLR7 was expressed at similar levels in PBMo and CBMo whereas TLR8 expression was significantly lower in CBMo compared to PBMo (Fig. 1A and B). Treatment of PBMo with resiquimod (R_848_) enhanced TLR7 expression levels, whereas administration of imiquimod (R_837_) lowered the TLR8 expression levels. TLR7/8 levels in CBMo remained unaltered after resiquimod R_848_ or imiquimod R_837_ treatment.

### TLR7/8 induced cytokine secretion

**Fig. 2:**
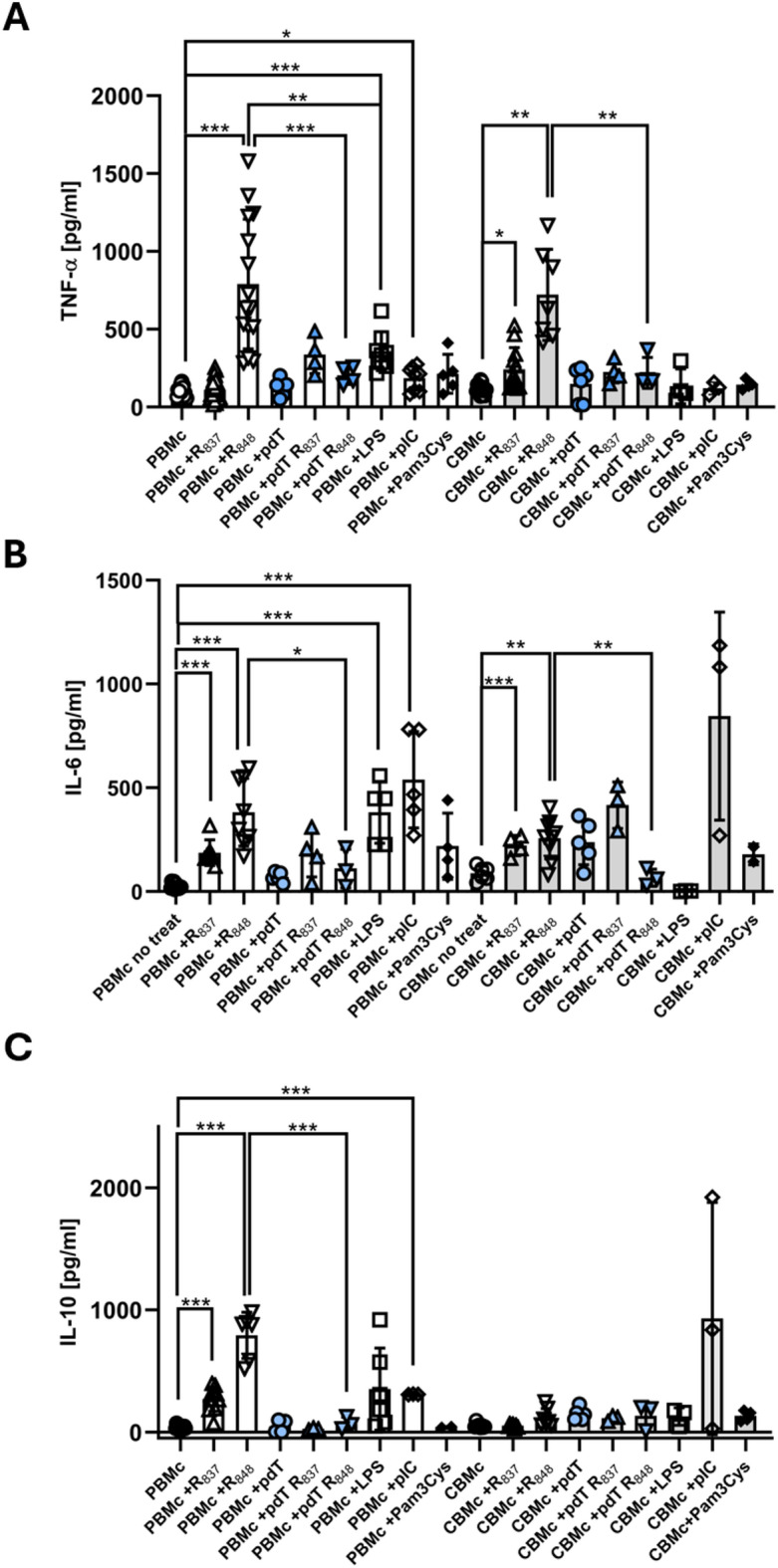
TLR7/8 mediated TNF-α, IL-6 and IL-10 secretion*.* TNF-α (**A**), IL-6 (**B**) and IL-10 (**C**) secretion by PBMc (white) and CBMc (grey) that were left untreated (−) or stimulated with imiquimod (R_837,_ 500 µM) or resiquimod (R_848,_ 40 µM) for 24 h. Cells were also pre-incubated with poly d:T (blue symbols; pdT, 0.5 µM) prior to stimulation. LPS (50 ng/ml), Pam3Cys (1 µg/ml) and pIC (100 ng/ml) were added for comparison (* *p* < 0.05, *p* < 0.05; **, *p* < 0.01; ***, *p* < 0.001; non-paired Student’s *t*-test).

Next, we analyzed whether stimulation with imiquimod (R_837_) and resiquimod (R_848_) led to TNF-α secretion by PBMc or CBMc (Fig. 2A). Resiquimod (R_848_) induced a strong secretion of TNF-α by both, PBMc or CBMc, whereas imiquimod (R_837_) led to significantly enhanced TNF-α secretion only in CBMc. The imiquimod (R_837_) induced TNF-α production was significantly lower than the resiquimod (R_848_) induced TNF-α production in CBMc. To analyze, which TLR accounts for the observed TNF-α production, we pre-incubated the cells with poly-d:T, (pdT) which has been reported to reduce TLR7 mediated signaling^13^. Adminstration of pdT abolished the TNF-α secretion in PBMc to both imiquimod (R_837_) and resiquimod (R_848_) and in CBMc to resiquimod (R_848_) (Fig. 1A, *p* < 0.05). No significant reduction in the percentage of the two monocyte populations was observed after imiquimod (R_837_) or resiquimod (R_848_) stimulation (Supplementary Fig. 2A). However, we detected an enhanced induction of apoptosis after addition of resiquimod (R_848_) in PBMo (Supplementary Fig. 2B). The TLR7/8 triggered TNF-α secretion was comparable to TLR3 (pI:C) and TLR4 (LPS) initiated TNF-α secretion in PBMc.

Secretion of TNF-α can provoke inflammation leading to tissue damage. To control hyperinflammatory reactions, stimulated Mo also react with secretion of modulatory and anti-inflammatory cytokines. To clarify this matter, we assessed the concentrations of IL-6 and IL-10 (Fig. 2B and C) in the supernatants of PBMc and CBMc. IL-6 secretion was induced to comparable levels by both TLR7/8 ligands imiquimod (R_837_) and resiquimod (R_848_) in PBMc and CBMc (Fig. 2B). Pretreatment with pdT significantly reduced the resiquimod (R_848_)—triggered IL-6 response but did not alter the imiquimod (R_837_) response in PBMc (Fig. 2B). In CBMc, pdT also reduced only the resiquimod (R_848_)—triggered IL-6 secretion. PBMc were also capable of IL-6 secretion after triggering TLR3 and TLR4. CBMc showed an enhanced level of IL-6 after TLR3 stimulation, although this reaction was not significant.

Imiquimod (R_837_) and even more so resiquimod (R_848_) induced a significant IL-10 secretion by PBMc but not by CBMc (Fig. 2C). The IL-10 response to both, imiquimod (R_837_) and resiquimod (R_848_) was abolished by pretreatment with pdT. In contrast, CBMc showed almost no reaction upon stimulation with TLR4/7/8 ligands. Only pIC caused an IL-10 secretion, indicating that the IL-10 response is inducible in CBMc.

### TLR7/8 signals in PBMo and CBMo via MAP kinases and NFκB

**Fig. 3:**
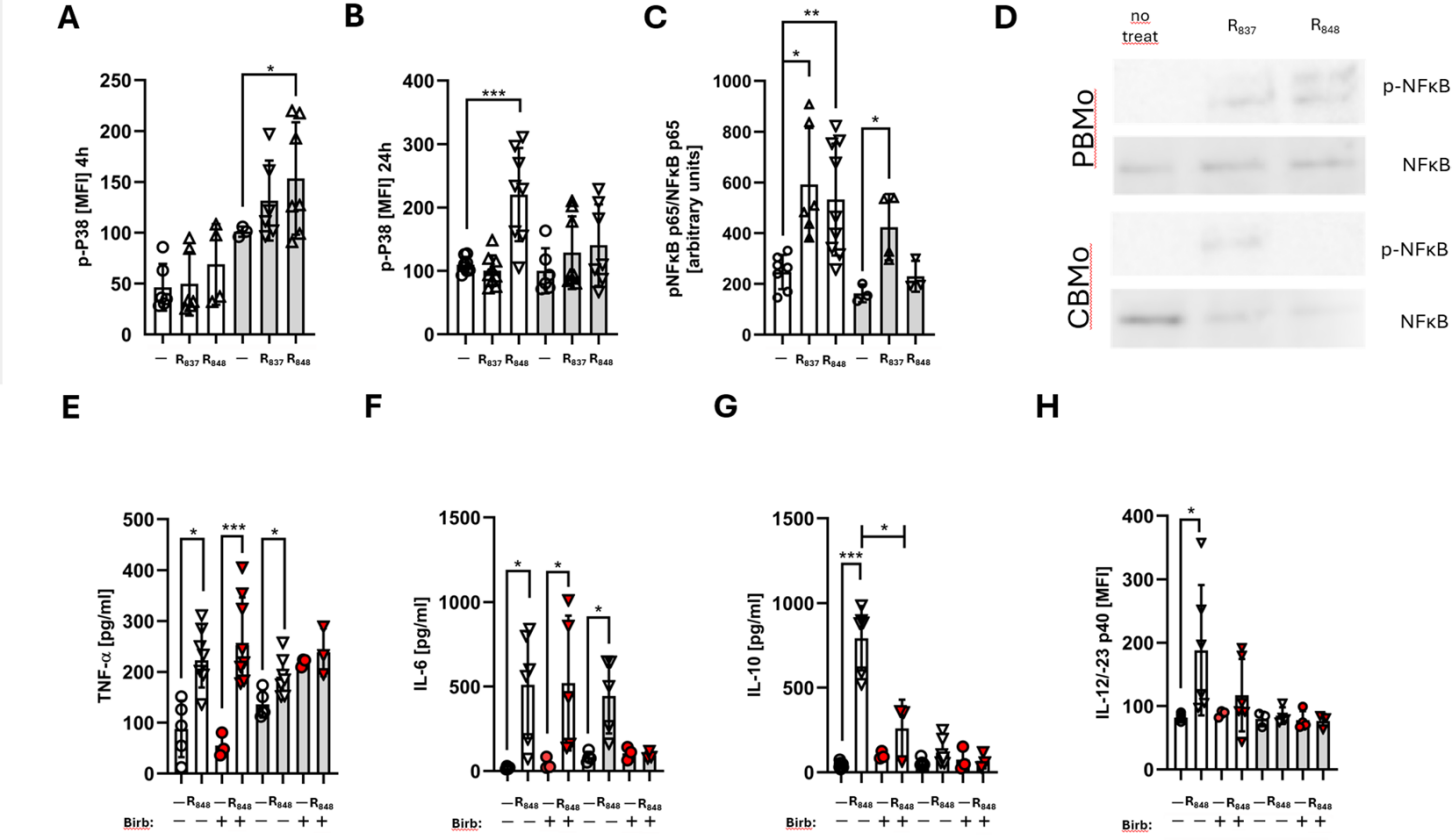
Signal transduction downstream of TLR7/-8. Activation of MAP kinase P38 was detected after 4 h (**A**) and 24 h (**B**) by FACS-based monitoring of the phosphorylated form after stimulation with indicated ligands (resiquimod (R_848_ 40 µM), imiquimod (R_837_ 500 µM). Activation of NFκB P65 (**C**) was assessed by detecting the phosphorylated form of the p65 subunit on immunoblots (**D**). Normalization was achieved by detection of the total NFκB P65 level. Secretion of TNFα (**E**), IL-6 (**F**) and IL-10 (**G**) in the absence or presence of BIRB-796 (Birb) was measured after stimulation with resiquimod (R_848_), Intracellular levels of IL-12/-23 p40 were assessed in the absence or presence of Birb (H, red symbols) under the indicated conditions (H). *, *p* < 0.05; **, *p* < 0.01; ***, *p* < 0.001; non-paired Student’s *t*-test).

Next, we aimed to identify the signal transduction pathway underlying cytokine production. To this end, we decided to study activated kinases via FACS analysis. Phosphorylated kinases were detected intracellularly in Mo located in the appropriate gate (Supplementary Fig. 1), which obviated mistakable detection of kinase activation in other cells. We started by characterizing the activation of the P38 MAP kinase in PBMo via monitoring the phosphorylation status (Supplementary Fig. 3, Fig. 3A, B). We first tested different concentrations namely 20, 100 and 500µM imiquimod (R_837_) and resiquimod (R_848_) in final concentrations of 2, 10 and 40 µM (Supplementary Fig. 3). Stimulation took place for short (5, 15, 30 and 60 min) and longer intervals (4 h, 24 h). Analyzing the highest concentration used (see graphs in Supplementary Fig. 3C), resiquimod (R_848_) induces phosphorylation quickly (5–30 min), followed by a phase of lower phosphorylation (60 min). At longer intervals (4–24 h) the phosphorylation is increasing again. Imiquimod (R_837_) exhibits a more delayed P38 phosphorylation, which is from 5 min to 24 h significantly higher than the control.

In CBMo, the P38 phosphorylation profile is different. Both, R_837_ and R_848_ seem to activate P38 in.

a delayed and attenuated manner compared to PBMo. Selecting the highest concentration of TLR7/8 ligands we tested the longer intervals and assessed significant imiquimod (R_848_) activation 4 h (CBMo) and 24 h (PBMo) after start of stimulation (Fig. 3A and B).

We also tested NFκB P65 activation upon stimulation. In PBMo, both imiquimod (R_837_) and resiquimod (R_848_) activated NFκB, whereas only imiquimod (R_837_) stimulated NFκB p65 in CBMo (Fig. 3C).

We next aimed to clarify, whether P38 or NFĸB, are prominent in signaling to cytokine production. Therefore, we co-incubated resiquimod (R_848_) -stimulated PBMc and CBMc with the p38 MAP kinase inhibitor BIRB-796 (doramapimod, Birb; Fig. 3E–H) and assessed the secretion of TNF-α (Fig. 3E), IL-6 (Fig. 3F), and IL-10 (Fig. 3G). Resiquimod (R_848_) -induced TNF-α secretion by PBMc was insensitive to Birb, but Birb lowered the TNF-α secretion in CBMc (Fig. 3E). A similar effect could be observed for IL-6 secretion. Resiquimod (R_848_)—induced IL-6 secretion was not affected by Birb in PBMc. In CBMc, Birb reduced the IL-6 secretion (Fig. 3F). In contrast, IL-10 secretion in PBMc was significantly reduced by Birb (Fig. 3G).

The results point to a P38 independent signaling regarding pro-inflammatory cytokines. IL-10, representing anti-inflammatory cytokines is P38 dependent at least in PBMc (Fig. 3G). Also, the NFκB dependent pathway can serve as an alternative signaling pathway to maintain pro-inflammatory cytokines. The production of IL-12/IL-23 p40 was found enhanced in PBMo after resiquimod (R_848_) stimulation (Fig. 3H), whereas no significant IL-12/-23 p40 was observed in CBMo. Pre-incubation of PBMo with the p38 MAP kinase inhibitor BIRB-796 abolished this effect (Fig. 3H).

### Type I IFN response engages IRF7 and is similar in PBMo and CBMo

**Fig. 4:**
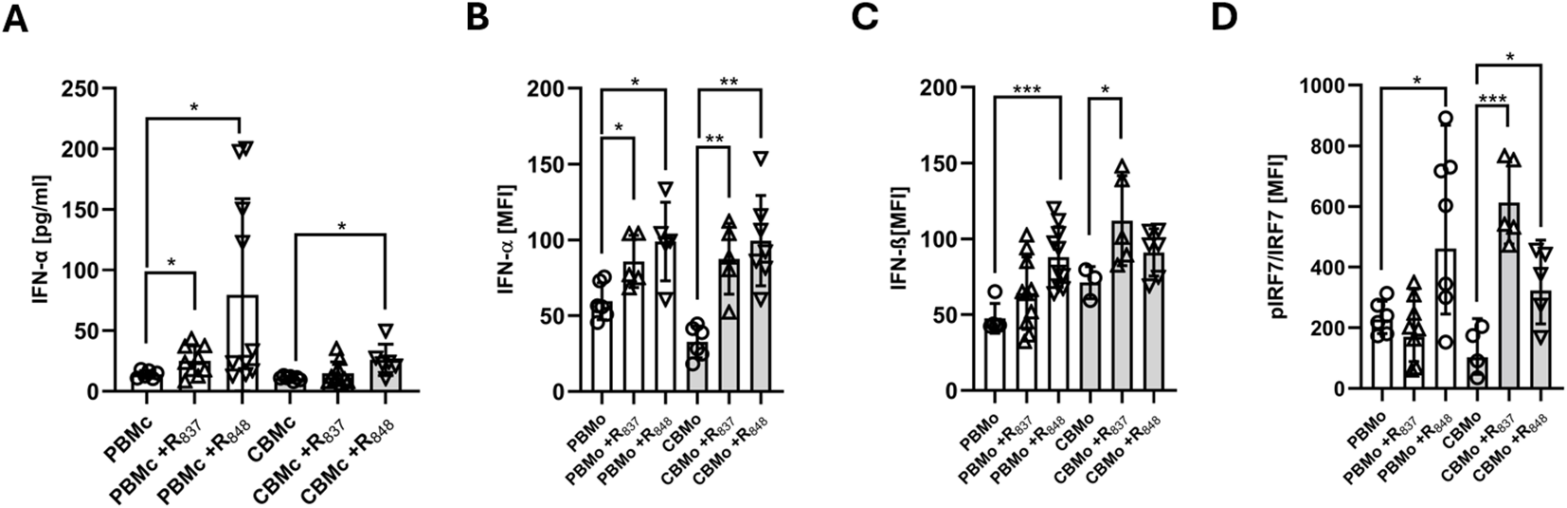
TLR7/8 stimulation induces secretion of IFN-α and -β. (**A**) PBMc (white) and CBMc (grey) were stimulated for 24 h with the indicated TLR ligands (resiquimod (R_848_)_,_ 40 µM), imiquimod (R_837,_ 500 µM) and IFN-α secretion was determined by ELISA. Intracellular staining for IFN-α (**B**) and -β (**C**) following stimulation of PBMo and CBMo with imiquimod (R_837_) or resiquimod (R_848_). (**D**) Intracellular staining of PBMo and CBMo for phosphorylated IRF7 after stimulation with imiquimod (R_837_) or resiquimod (R_848_). *, *p* < 0.05; **, *p* < 0.01; ***, *p* < 0.001; non-paired Student’s *t*-test.

TLR7/8 stimulation is known to trigger anti-viral responses^7^. We, therefore, assessed the expression and secretion of type I interferon (IFN-α) comparatively by PBMc and CBMc (Fig. 4A). Both ligands, imiquimod (R_837_) and resiquimod (R_848_) caused secretion of IFN-α in PBMc whereas only resiquimod (R_848_) induced secretion of IFN-α by CBMc (Fig. 4A).

To confirm that monocytes were the source of the secreted cytokine we performed intracellular staining and detected increased intracellular IFN-α after imiquimod (R_837_) and resiquimod (R_848_) stimulation both in PBMo and CBMo as well as intracellular IFN-β after stimulation of PBMo with resiquimod (R_848_) and CBMo with imiquimod (R_837_) (Fig. 4B and C). Next, we tested phosphorylation of the transcription factor IRF-7, likely activated by imiquimod (R_837_) and resiquimod (R_848_) treatment. In PBMo, only resiquimod R_848_ stimulation led to an increase in the phosphorylated form of IRF-7, whereas in CBMo, both imiquimod (R_837_) and resiquimod (R_848_) triggered significant phosphorylation of IRF-7 (Fig. 4D).

### TLR7/8 stimulation interferes with the phagocytic capacity of monocytes

**Fig. 5:**
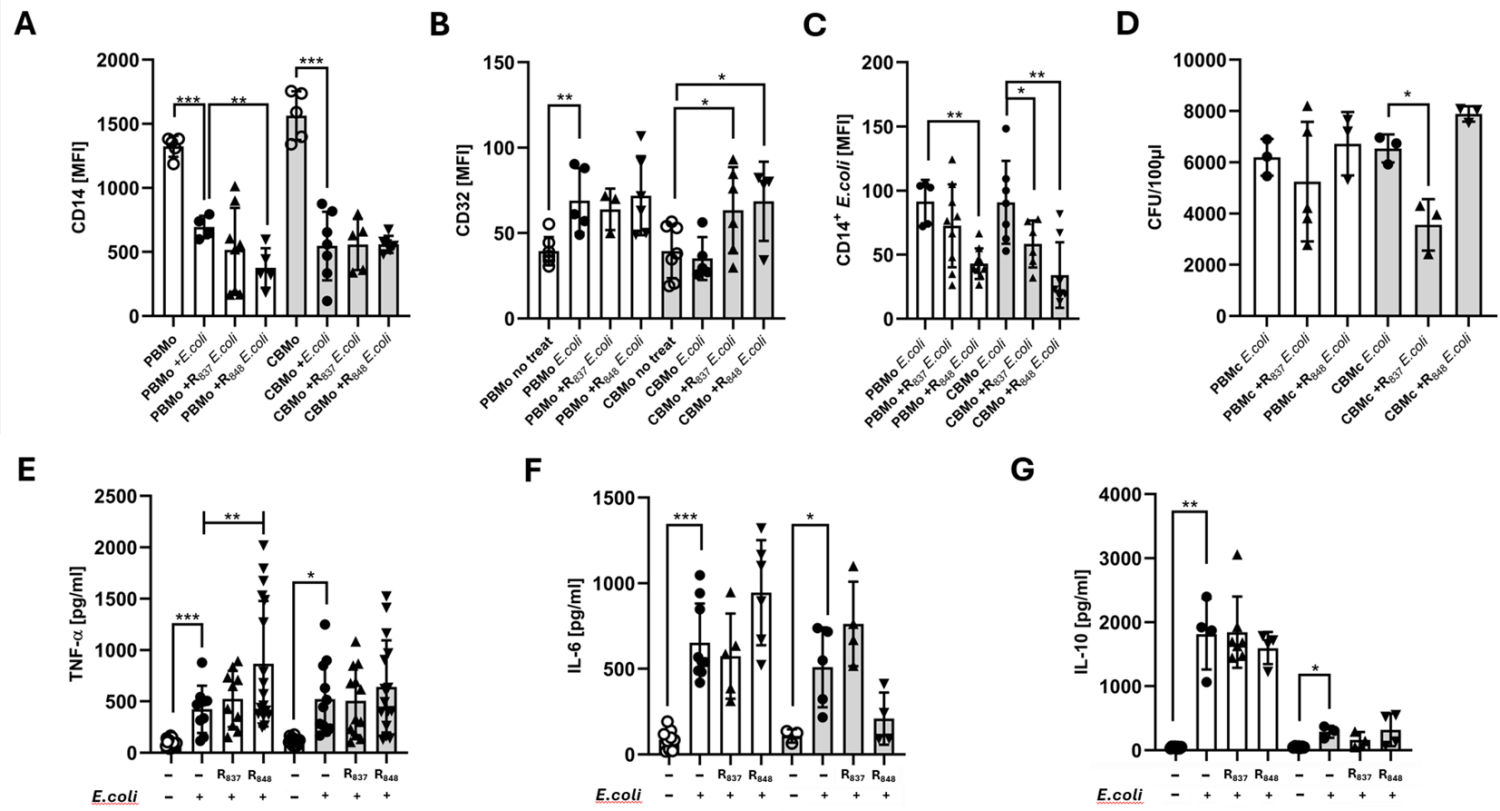
Influence of TLR7/8 stimulation on the peri-phagocytotic reactions of monocytes*.* PBMo and CBMo were prestimulated for 24 h with (resiquimod (R_848,_ 40 µM), imiquimod (R_837,_ 500 µM) and subsequently co-cultured with *E. coli* for 24 h (MOI 25). The CD14 (**A**) and CD32 (**B**) mean expression was assessed. The phagocytic index (**C**) was assessed as described in material and methods. (**D**) The number of viable *E. coli* was determined by serial dilution and plating (plaque forming units, CFU).Secretion of TNF-α (**E**), IL-6 and IL-10 (**G**) from TLR7/8 prestimulated and *E. coli* co-cultured PBMc and CBMc was detected (MOI 25; *, *p* < 0.05; **, *p* < 0.01; ***, *p* < 0.001; non-paired Student’s *t*-test; blunt ended bar represent analysis via two-way ANOVA; **, *p* < 0.01).

To comparatively analyze the influence of TLR7/8 stimulation on the phagocytotic and bactericidal capacity of PBMo and CBMo, we stimulated the primary monocytes with imiquimod (R_837_) or resiquimod (R_848_) prior to the addition of the opportunistic pathogen *E. coli*. First, we determined the expression of the surface markers CD14 and CD32 (Fig. 5A and B), which are functional in *E.coli* phagocytosis. In PBMo resiquimod (R_848_) further decreased the *E.coli* induced reduction of CD14. In CBMo no such effect was observable. In contrast, imiquimod (R_837_) and resiquimod (R_848_) enhanced the CD32 expression synergistically with *E.coli* challenge in CBMo (Fig. 5B). The results could be interesting in regard to the opsonization state of *E.coli*, since CD14 is phagozyting non-opsonized *E.coli*, while CD32 is functional for opsonized *E.coli.*

PBMo exhibited a decrease in their phagocytotic capacity after preincubation with resiquimod (R_848_), whereas both, imiquimod (R_837_) and resiquimod (R_848_) reduced the uptake of *E. coli* by CBMo (Fig. 5C). The bactericidal capacity was only significantly reduced after pre-stimulation of CBMo with imiquimod (R_837_) (Fig. 5D).

Moreover, we tested the release of TNF-α, IL-6 (Fig. 5E and F) and IL-10 (Fig. 5G) in the same experiments. Co-culture with *E. coli* increased the secretion of TNF-α and IL-6 by both, PBMc and CBMc (Fig. 5E and F). In addition, resiquimod (R_848_) enhanced the TNF-α secretion by *E. coli*-infected PBMc, whereas neither imiquimod (R_837_) nor resiquimod (R_848_) influenced the TNF-α secretion by CBMc. We observed no synergistic effects of TLR7/8 stimulation together with *E.coli* infection in regard to IL-6 and IL-10 secretion (Fig. 5F, G).

## Discussion

TLR7 and TLR8 are PAMP receptors that sense ssRNA from pathogens in general and viruses in particular^14^. The finding that both PAMP receptors share not only a great sequence homology but sense imidazo [4,5-c] quinoline molecules such as R_837_, was established by pharmacological approaches that initiated the signaling cascade without the use of infectious viral ssRNA^15^. Since it is unclear whether TLR7 and TLR8 are equally functional in neonatal and adult monocytes^16^, we comparatively analyzed the expression patterns of TLR7 and 8. The correlation between neonatal TLR8 expression and signaling was described in several publications. The first study showed that expression of TLR7 and TLR8 peaked at gestational week 33–35^17^. Others described a similar expression of both TLR7 and 8 in monocytes on mRNA and protein level in term newborns^18,19^. In contrast, reduced expression of TLR8 or a reduced fraction of TLR8-positive CBMo was reported^20^. Low expression of TLR8 in monocytes was shown to be associated with a reduced early cytokine response, delayed initiation of an adaptive Th1 immune response during RSV infection and control of viral replication^21^.

Expression of TLR7 was found to be only slightly different in naive CBMo and PBMo, but TLR8 was significantly stronger expressed in PBMo. Together with our findings, that imiquimod (R_837_) per se induced a stronger TNF-α secretion in CBMc only, and P38 activation is attenuated in imiquimod (R_837_)—stimulated CBMc and PBMc, the interpretation is conceivable that the induction of an adaptive Th1 responses by CBMo relies more on resiquimod (R_848_) and the TH2 response on imiquimod (R_837_).

TNF-α and IL-1ß secretion was shown to be similarly inducible in adults and newborn monocytes after TLR8 stimulation^22^. A selective inducibility of TNF-α by TLR7/8 stimulation was reported for imiquimod (R_837_)^19^. Here, we collected evidence for a reduced cytokine response via TLR7 signalling^19^. Our findings are supported by reports, which described a marginal cytokine response after imiquimod (R_837_) stimulation in monocytes. Moreover, experimental results suggest that the TLR7 and TLR8 signaling cascades are competing for the recruitment of downstream factors for cytokine response, which was shown by imiquimod (R_837_)—driven inhibition of TLR8 signaling^23^. In contrast, some groups did not find different cytokine responses upon TLR7/8 stimulation^24^.

Several studies support the hypothesis that immune cells trained by TLR7/8 pre-stimulation show a modulated outcome to nosocomial bacterial infection. For RSV infections, epidemiological data show a high frequencey of co-infections with *streptococcus* or *pneumococcus* in young children^25,26^. Infections with influenza virus in newborns are worsened by gram-negative bacterial (*E. coli*) co-infections^27^. Resiquimod (R_848_) was shown to be a strong inducer of IFN-α, TNF-α, IL-6 and IL-10 secretion independent of the influenza virus or *pneumococcus* infection status in PBMCs from pre-school children^28^. On the other hand, exposure to bacterial membrane vesicles was shown to reduce the damage caused by viral entry^29^. Also, imiquimod (R_837_) was shown to down-regulate co-receptors engaged in HIV-1 entry^30^. The incidences of NEC and bacterial sepsis were reported to be reduced after the SARS-CoV2 pandemia^31^.

Therefore, the second experimental approach employed in this manuscript, aimed to study the modulating effect of an initial TLR7/8 stimulation followed by *E. coli* co-culture. Studies which examine the role of TLR7/8 ligands on the phagocytic capacity of monocytes have rarely been reported. In anti-tumor therapies, the administration of resiquimod (R_848_) together with SIRP-1 inhibitors reinforces phagocytic activities^32^. This is in line with our observation of a reduced phagocytic index and phagocytic capacity for PBMo after pre-incubation with resiquimod (R_848_), but not with imiquimod (R_837_), which is more specific for TLR7. Interestingly, the phagocytic capacity was reduced in CBMo after stimulation with both imiquimod (R_837_) and resiquimod (R_848_). These findings indicate differences in the downstream effects of TLR signaling in CBMo and PBMo.

We published recently that infection with *E. coli* reduced CD14 expression in PBMo as well as CBMo due to binding of *E. coli* or shedding of the receptor^33^. Surprisingly, TLR7/8 ligands reduced the CD14 density on monocytes and this effect may explain the reduced phagocytic capacity. Further experiments are needed to characterize, why PBMo and CBMo react differently to imiquimod (R_837_) and resiquimod (R_848_)_._ Although both stimuli reduce CD14 expression, PBMo exhibited reduced phagocytic capacity after resiquimod (R_848_) stimulation. The same effect was observed for CBMo upon imiquimod (R_837_) exposure. In an osteomyelitis model, the enhanced neutralization of *Staphylococcus aureus* by resiquimod (R_848_) was accompanied by an increase in the pro-inflammatory response^34^. Importantly, a reduced bacterial load was found in imiquimod (R_837_) pre-stimulated CBMo (Fig. 5B), which correlated with a reduced phagocytic index. It is speculative but tempting to think about an imiquimod (R_837_) treatment to reduce opportunistic bacterial infections in neonates. However, the limitations of our *in-vitro* data need animal models to test this theory.

Comparable results were obtained for the pleiotropic cytokine IL-6, but synergistical effects could not be observed. Recent work documented with spike activation assays revealed a synergistic increase of IL-6 secretion after combined LPS and resiquimod (R_848_) stimulation^35^.

The IL-10 secretion differed dramatically between PBMc and CBMc. Whereas TLR7/8 stimulation triggered IL-10 release in PBMc, it failed to do so CBMc. Also, the synergistic effect of resiquimod (R_848_) and *E. coli* co-culture on TNF-α secretion in PBMc, was not observed in CBMc.

High secretion of type 1 IFN as an anti-viral response was reported for pDCs^18^. We assessed intracellular IFN-α and IFN-β protein levels to prevent that type 1 IFN in the cell culture supernatant may be derived from contaminating DCs or T-cells. Like IL-6 and TNF-α, IFN-α production is caused by resiquimod (R_848_) in PBMo and by imiquimod (R_837_) and resiquimod (R_848_) in CBMo. The inducibility of IFN-α in CBMo by both ligands suggests a mechanism, which is less conditioned by TLR8 signaling. The IFN-β induction by TLR7/8 ligands is restricted to PBMo, whereas CBMo showed no response. Further experiments need to clarify whether IRF proteins, signal transducers engaged in the production of type I IFN are selectively modulated in CBMo. In this context, IRF1 and IRF5 could be of special interest. Recently, IL-10 was shown to negatively regulate IRF signaling which downmodulates IFN responses^36^. The absence of the IL-10 response in CBMo is a putative strategy to privilege the anti-viral defense accepting a sustained state of inflammation. Future experiments should test this hypothesis by supplementing CBMo with additional IL-10. The MAP kinase and NFκB pathway are active both in CBMo and PBMo. IRF5 cooperates with NFκB^37^ and a shortcoming of IRF5 could modulate NFκB in CBMo.

A special intricacy of studying TLR7/8 is the negative competition of their signaling. TLR8 downregulation or complete genetic knock-down leads to overexpression of TLR7 which leads to auto-immune reactions. Furthermore, TLR8 is activated by bacterial DNA^38^ which makes it difficult to interprete our results regarding mixed challenges with *E. coli* and TLR7/8 stimuli.

We could show a P38 activation in Mo for different time intervals (Fig. 3A, B; Supplementary Fig. 3). In PBMo, resiquimod (R_848_) induces phosphorylation after 5 min followed by a second wave of phosphorylation at 4 h PBMo and 24 h (CBMo). Also, the kinetic pilot experiments cannot rule out that imiquimod (R_837_) leads to a P38 activation to earlier time points. This finding is challenging for dissecting the signal crosstalk initiated by bacterial and TLR7/8 ligands. Recent work indicated that IRF7 activation in Mo is low after 1 h of stimulation and is fully detectable 20 h later^18^. With exception of the P38 activation analysis, we decided to monitor cytokine release and production to late time points. We have shown in previous studies, that P38 activation occurred after LPS and *C.albicans* stimulation^39^. In this context, working with non-purified Mo is limiting the interpretations of signal mechanisms. The bi-phasic activation of P38 upon resiquimod (R_848_) stimulation is potentially due to the involvement of other cells than Mo, such as T-cells or dendritic cells. P38 can be initiated by TLR7/8 signaling quickly. Interaction with other cells can start the second activation in *trans*.

Together, TLR7/TLR8 signaling in neonatal and adult monocytes differs markedly. CBMo display an intact inflammatory and antiviral response but lacks IL-10 secretion, resulting in a pro-inflammatory phenotype. The present findings highlight age-dependent differences in the TLR pathways relevant for the neonatal antimicrobial host defense and infection susceptibility.

## Material and methods

### Patient samples

The experimental procedure presented here was approved by the Ethics Committee of Aachen University Hospital (Permission No: EK150/09, Oct. 6, 2009). For minors, informed consent was obtained from the parent(s) and/or legal guardian for study participation. All adult participants gave written informed consent prior to having venous blood samples taken. All methods were performed in accordance with the relevant guidelines and regulations.

Only neonates delivered spontaneously and showing no signs of infection, were accepted for this study. Health status was determined by examination of white blood cell count, Interleukin-6 (IL-6), C-reactive protein and clinical status. The results of clinical investigations were discussed with gynecologists and midwives before taking samples. Immediately after cord ligation, umbilical cord blood samples were placed in heparin-coated tubes (10 IU/ml blood). Neither mothers in whom the amnion had become infected or labour had been prolonged (> 12 h) nor SGA neonates (small for gestational age) nor preterm infants before 36 weeks of gestation were accepted for the study.

In this study we collected umbilical cord blood from 25 donors in total. Additionally, we collected sex matched samples from 30 adult donors.

### Reagents and antibodies

Primary antibodies were purchased by Thermo-Fisher-Scientific (NRW, Germany). The surface antigen antibodies anti-CD14 (clone MEM-18, #21270143, Immunotools, Niedersachsen, Germany), anti-CD32 (clone 3D3, #552884, Becton Dickinson, Switzerland) have been used according to the manufacturers` recommendations. Antibodies specific for intracellular epitopes, namely, anti-IFN-α (clone 7N4-1, 1:100 diluted, #560088, Becton–Dickinson, Switzerland), anti-IFN-ß (clone A1(IFNb), 1:200 diluted, #BMS1044FL), anti-TLR8 (clone 44C143, 1:200 diluted, #MA5-16194), anti-TLR7 (clone 533707, 1:100 diluted,, R&D, Minnesota, USA), anti-IRF7 (clone 23GB2925,1:100 diluted, # MA5-52510), anti-phospho-IRF7 (Ser 470, Ser 471, 1:100 diluted, # BS-3196R), anti-phospho P38 (clone 4NIT4KK, Thr 180, Tyr 182, 1:500 diluted, # 25-9078-42), anti TNF-α (clone Mab11, 1:100 diluted,. # 404-7349-42), anti-NFĸB P65 (clone D14E12, 1:500 diluted, #8242, Cell Signaling Technology, Leiden, the Netherlands) and anti-phospho-NFĸB P65 (clone 93H1, Ser 536, 1:1000 diluted, #3033, Cell Signaling Technology, Leiden, the Netherlands),

The P38/JNK-2 inhibitor Birb 796 (Doramapimod, # S1574, Sellekchem, NRW, Germany) was added at a final concentration of 1 µM^40,41^. Propidiumiodide (PI, BMS500PI), Kanamycin (J67354.AD, 50 mg/ml) and IPTG (R1171) was purchased fromThermo-Fisher-Scientific (NRW, Germany). LPS was from Thermo Fisher Scientific (00-4976-93, NRW, Germany), Pam3Cys and poly I:C (pIC) from InvivoGen (Toulouse, France, tlrl-pms, vac-pic**).**

### Isolation of mononuclear cells

For this study we collected umbilical blood from 25 donors. Adult blood samples were taken from 30 donors, which were sex-matched and aged 20–50 years. Mononuclear cells were isolated by Ficoll density gradient from whole blood samples and cultivated in RPMI 1640 medium (Gibco, Thermo-Fisher-Scientific, NRW, Germany) supplemented with FCS (Gibco, Thermo-Fisher-Scientific, NRW, Germany, 10% v/v) under standard conditions as already published^33,42^. In this medium, we seeded 1 × 10^6^ cells/ml prior to stimulation.

Mononuclear cells were designated as PBMc and CBMc, respectively. Both were used in ELISA assays. For FACS based assays Mo were gated from the mononuclear population and designated as PBMo and CBMo respectively (Supplementary Fig. 1). The accuracy of the Mo gate was checked by CD14 staining (Supplementary Fig. 1).

### Stimulation of PBMc and CBMc and treatment with inhibitors

Activation of TLR7/8 was initiated for an interval of 4 to 24 h with imiquimod (R_837_) and resiquimod (R_848_)_,_ respectively. Both base analogues were purchased from Invivogen (Toulouse, France, tlrl-imqs-1, tlrl-r848-1). With exception of the dose kinetic studies (Supplementary Fig. 3), we used 500 µM imiquimod (R_837_) and 40 µM resiquimod (R_848_). To compare stimulation efficacy, PBMc and CBMc were also stimulated with LPS (50 ng/ml), Pam3Cys (1 µg/ml) or pIC (100 ng/ml). The inhibitor Birb 796 (Birb) was added 30 min before TLR7/8 stimulation. Samples with Birb 796 were compared to a control using DMSO. For modulating the TLR7/8 stimulation, poly d:T (pdT, #tlrlr-pt17, Invivogen, Toulouse, France) was added together with R_837_ and R_848_ in a final concentration of 0.5 µM ^13,16^. Indicated samples were infected with *E. coli* with or without combination of imiquimod (R_837_) and resiquimod (R_848_) for an additional 24 h. Cells were fixed in final concentration of 1% paraformaldehyde (PFA) in PBS for 1 h and permeabilized with permeabilization buffer (#0083356, Thermo Fisher Scientific NRW, Germany). Afterwards cells were washed with the permeabilization buffer and subsequent primary antibody incubation took place at room temperature (RT). For intracellular staining with non-fluorochrome labelled primary antibodies, cells were permeabilized and stained as described above. The secondary anti-rabbit antibody (Fab fragment #A66788, Thermo Fisher Scientific NRW, Germany) was added in a dilution of 1:500 and incubated for additional 30 min at RT. Prior to FACS analysis all samples were washed once with PBS.

### ELISA

The following ELISA kits, namely IL-6 ELISA (#31670069 Immunotools, Niedersachsen, Germany), IL-10 ELISA (#BMS215-2TEN, Thermo-Fisher-Scientific, NRW, Germany), TNF-α ELISA (#BMS223-4, Thermo-Fisher-Scientific, NRW, Germany) and IFN-α ELISA (Invitrogen) were used according to the manufacturers` recommendations.

### Phagocytosis assay

The phagocytosis index (CD14^+^GFP^+^ Mo %: CD14^+^ Mo %) and the phagocytic capacity (mean fluorescent intensity (MFI) of CD14^+^ monocytes) were assessed by flow cytometry after 24 h p.i. (post infection). The cultivation of *E. coli* and the genetic background is published elsewhere^42^. The *E. coli* strain DH5α, an encapsulated K12 laboratory strain, carrying the green fluorescent protein (*gfp*)-mut2 gene (*E. coli*-GFP) was a gift of Prof. Dr. C. Gille (University of Heidelberg). In brief, we cultivated GFP expressing *E.coli* in LB-medium, supplemented with Kanamycine (50 µg/ml) and IPTG (1 mmol/l). Growth was allowed until an OD of 600 was reached. Bacterial suspension was centrifuged at 300xg for 10 min and the pellet resuspended in PBS. For bacterial phagocytosis assays a multiplicity of infection of 25 (MOI 25) was utilized.

### Neutralization assay

In brief, *E. coli* infection took place for 1 h at standard conditions. Afterwards, the medium was replaced by medium containing kanamycine (50 µg/ml) for additional 3 h. PBMc and CBMc were washed in PBS prior to disruption by addition of 0.1%v/v Triton-X 100 and vigorous shaking. After centrifugation, the pellet was resuspended in LB and applied to LB agar plates (PO5309A, Thermo-Fisher-Scientific, NRW, Germany). After 24 h at 37 °C plaques were counted.

### Immunoblot analysis

For checking FACS based intracellular staining, we performed immunoblot analysis for NFĸB P65 / phospho-NFĸB P65 expression. To this end, we removed lymphocytes from PBMc and CBMc cultures by using a negative selection kit from Mylteni Biotech (Mylteni Biotech, NRW, Germany). In brief, isolated PBMc and CBMc were incubated with a magnetic-bead coupled mix of antibodies specifically binding to T-cells, B-cells, dendritic cells and NK-cells according to protocols published by the supplier (Mylteni Biotech, NRW, Germany). These cells remained bound to a matrix, whereas the flow through contained Mo with a purity > 90% (See also Supplementary Fig. 1). Afterwards, the purified Mo were washed twice in PBS before being lysed with RIPA buffer (150 mM Sodium chloride, 1% v/v NP-40, 50 mM pH 8 Tris/HCl, 0,5% v/v Sodium deoxycholate, 0,1% v/v SDS). The crude cell lysate was centrifuged two times at 4 °C. The 4-time concentrated Laemmli buffer was added to the supernatants. After boiling for three minutes, the lysates were deployed to SDS-PAGE and transferred to nitrocellulose membranes (according to standard protocols of Lämmli and Khyse Anderson). For imaging and quantification, a LAS 3000 imager (Fujifilm, NRW, Germany) combined with the Multi-Gauge software (Fujifilm, Düsseldorf, Germany) was used.

### Detection of apoptosis by hypodiploid nuclei

Hypodiploid nuclei were assessed according to the method described by Nicoletti et al.^43^. CBMC and PBMc were isolated and preincubated with bacteria for 1 h. After removal of free bacteria, CBMc and PBMc were incubated for 24 h. We then slowly re-suspended the CBMc and PBMc in 2 ml of − 20 °C ethanol 70%, with continuous vortexing, followed by storage for 4 h at − 20 °C. Cells were washed twice in PBS, re-suspended in 50 μl PBS containing 13 kunitz units RNase (EN0531, DNase-free, Thermo-Fisher-Scientific, NRW, Germany), and incubated for 15 min at 37 °C. We then added 180 μl of PI solution (70 μg/ml in PBS). The analysis was done within 20 min of incubation.

### Statistical analysis

Results are expressed as mean + /- standard deviation. Error bars represent standard deviations (SD). The pilot experiments in Supplemental Fig. 3 show mean values and standard error of mean (SEM). Values of *p* < 0.05 were considered significant. Analyses were done with statistical software performing student`s t-test and two-way ANOVA. Experiments where N = 3 were tested according to Mann–Whitney for significant difference. Data which did not pass a test for Gaussian distribution were tested with a Kolmogorov–Smirnov test as provided by Graph Prism Pad Software Statistical Package, La Jolla, CA 92037 USA.

## Supplementary Information

Below is the link to the electronic supplementary material.

Supplementary Material 1

Supplementary Material 2

Supplementary Material 3

Supplementary Material 4

Supplementary Material 5

Supplementary Material 6

Supplementary Material 7

Supplementary Material 8

Supplementary Material 9

## Data Availability

All data supporting the findings of this study are available within the paper and its Supplementary Information.
