# Supplementary material for: TLR7/8 signaling balances cytokine responses in neonatal monocytes
Source: Sci Rep. 2026 Apr 13;16:12202. doi: 10.1038/s41598-026-46534-6 (PMC13076771; doi:10.1038/s41598-026-46534-6)
Supplement: Supplementary file 1 — Supplementary Material 1 [file 41598_2026_46534_MOESM1_ESM.docx]

**Supplementary Figures**

**Supplementary Figure 1**

**
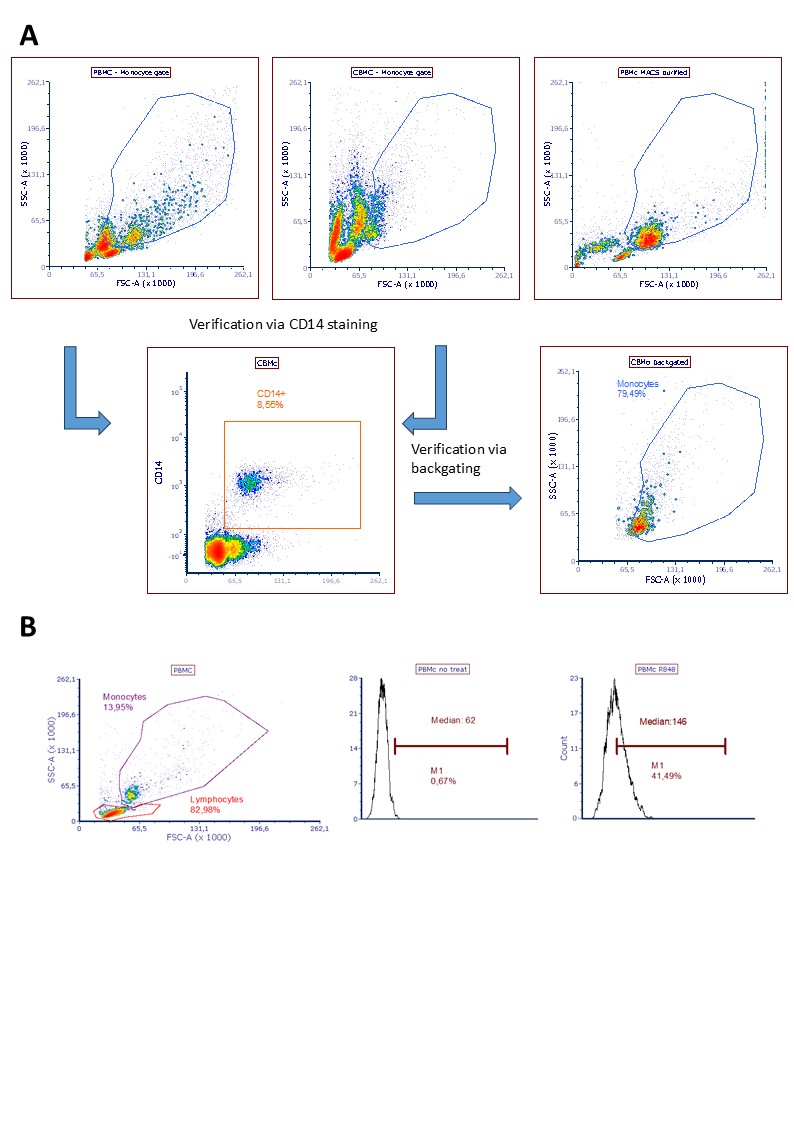
**

**Supplementary Figure 1: Gating strategy.** Upper panels show position of the Mo Gate in PBMc (A, left), CBMc (A, middle) and negatively purified Mo (A, right). Lower panels depict the verification of the Mo gate by CD14 staining and backgating, The PBMo in the Mo Gate were subjected to TLR7/8 stimulation and stained for IFN-α (B).

**Supplementary Figure 2**

**
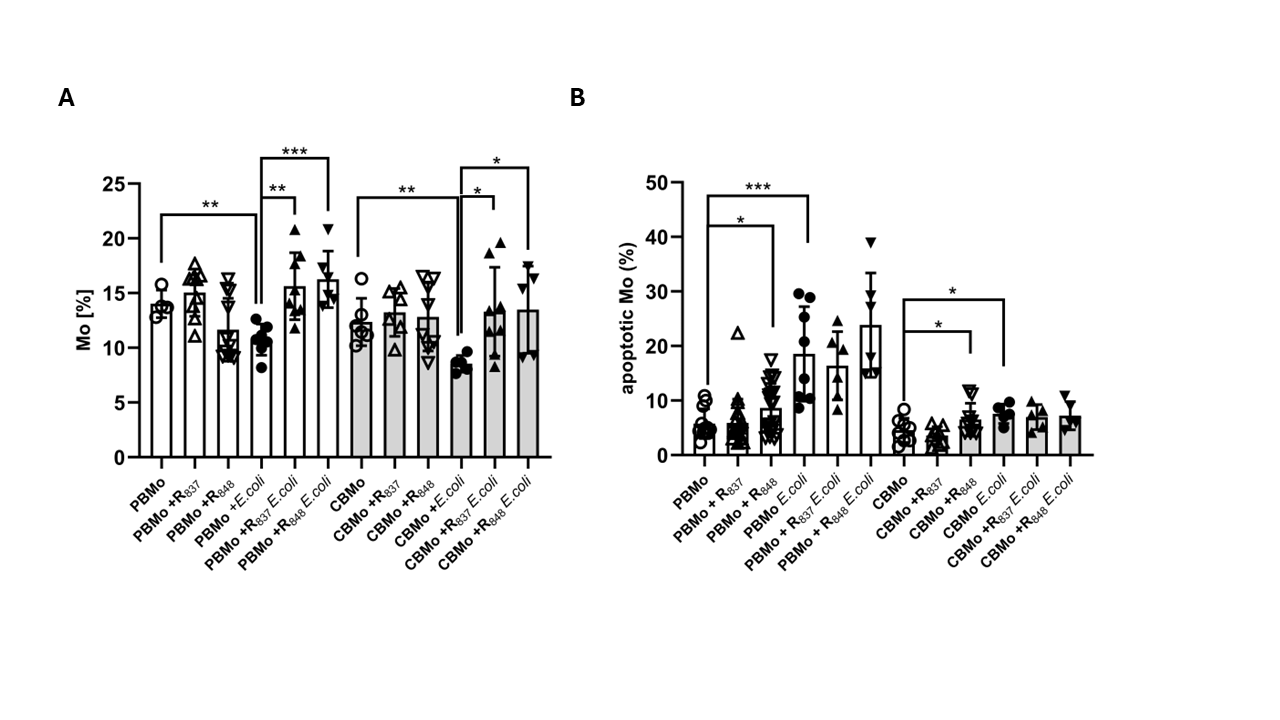
**

**Supplementary Figure 2: Proportion of Mo in PBMCs and CBMCs and induction of apoptosis.**

The percentage of Mo was assessed (A) and the percentage of Mo exhibiting a hypodiploid subG1 DNA (B) resembling apoptotic cells (* *p* < 0.05, ** *p* < 0.01, *** *p* < 0.001, *p* represents non-paired Student’s *t*-test)

**Supplementary Figure 3**

**
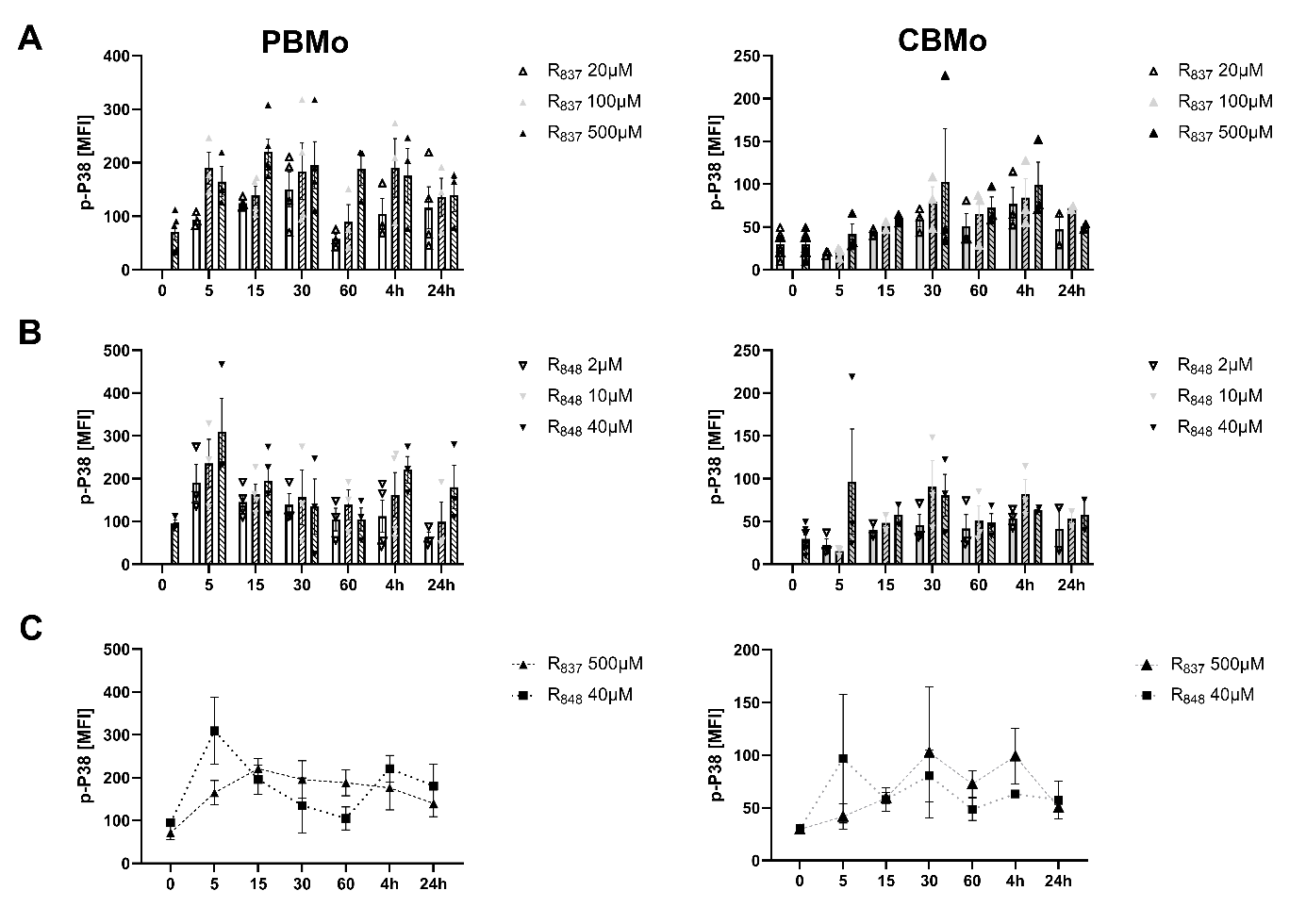
**

**Supplementary Figure 3: TLR7/8 ligands dose response time course in regard to P38 phosphorylation.**

The mean phosphorylation of P38 was assessed in time intervals indicated for PBMo, treated with indicated concentrations of imiquimod (R_837_) (A) and resiquimod (R_848_) (B). Corresponding experiments were also given for CBMo. In (C) the summarized results of the highest concentrations of TLR7/8 ligands are presented. The graphs show N=3 experiments for 3 donors, respectively. The error bars represent SEM.
