# Supplementary figures and images for: TLR7/8 signaling balances cytokine responses in neonatal monocytes
Source: Sci Rep. 2026 Apr 13;16:12202. doi: 10.1038/s41598-026-46534-6 (PMC13076771; doi:10.1038/s41598-026-46534-6)

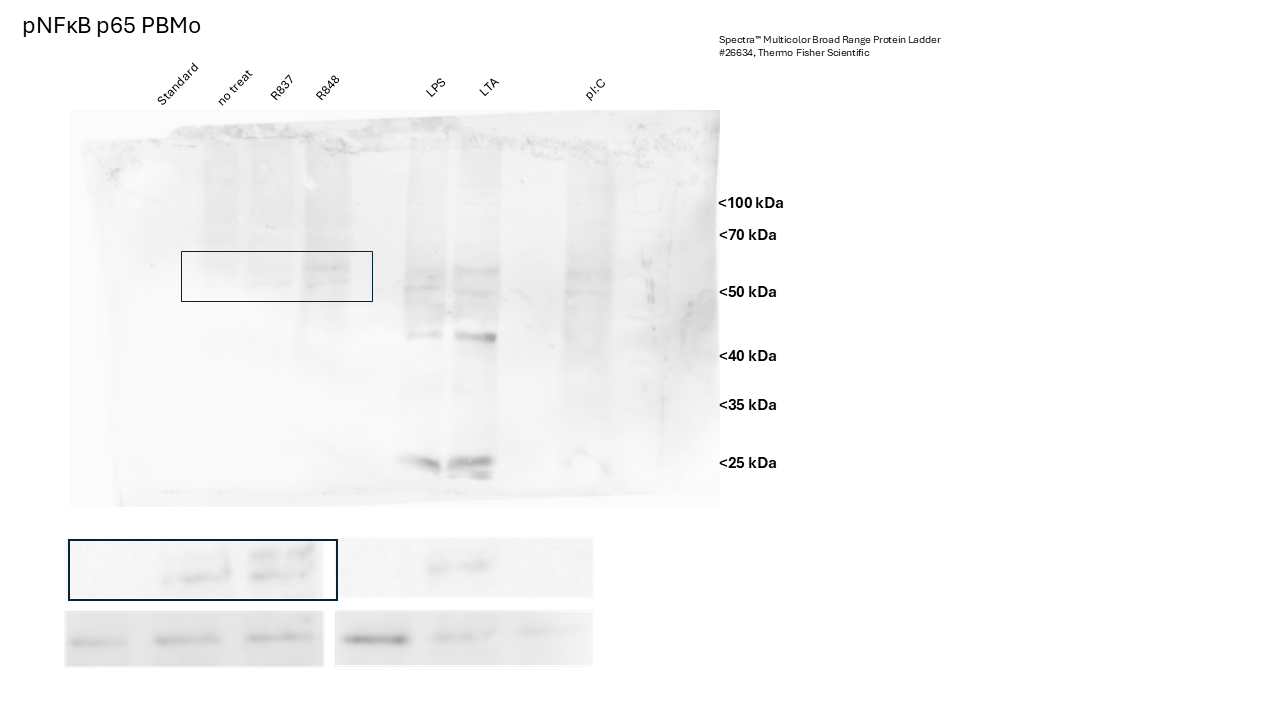

Supplement: Supplementary file 2 — Supplementary Material 2 [file 41598_2026_46534_MOESM2_ESM.png]

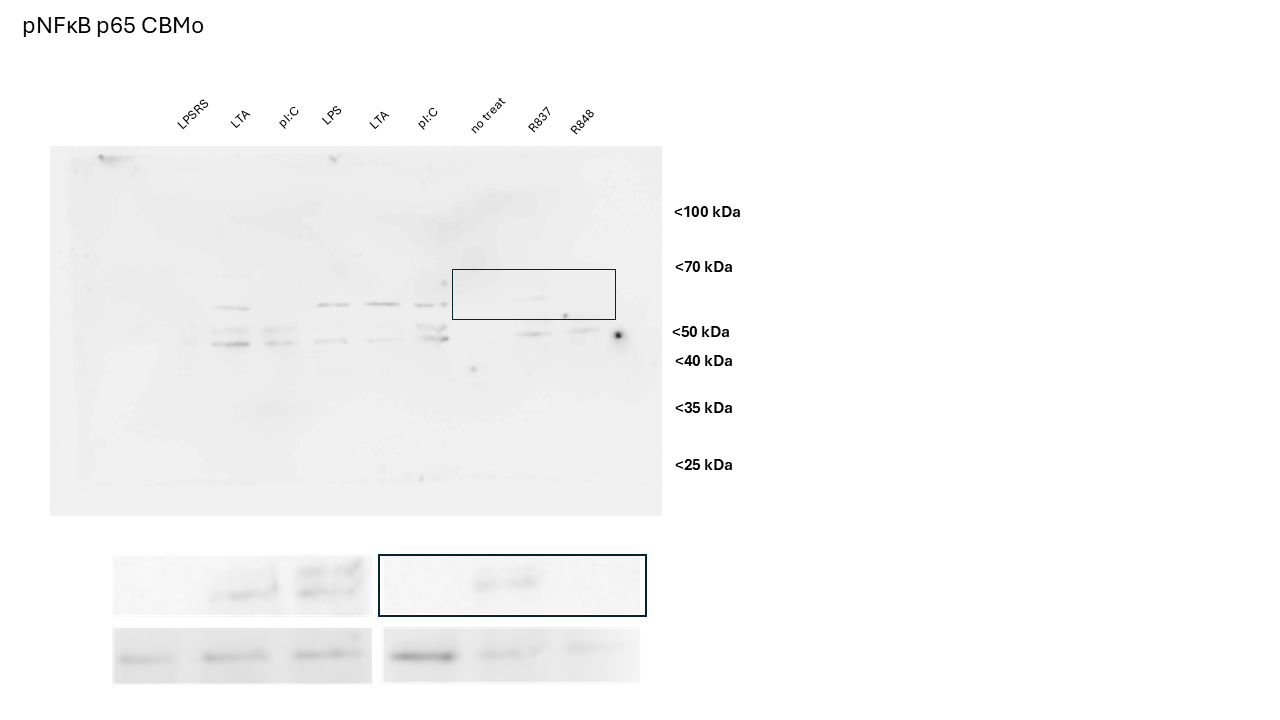

Supplement: Supplementary file 4 — Supplementary Material 4 [file 41598_2026_46534_MOESM4_ESM.png]

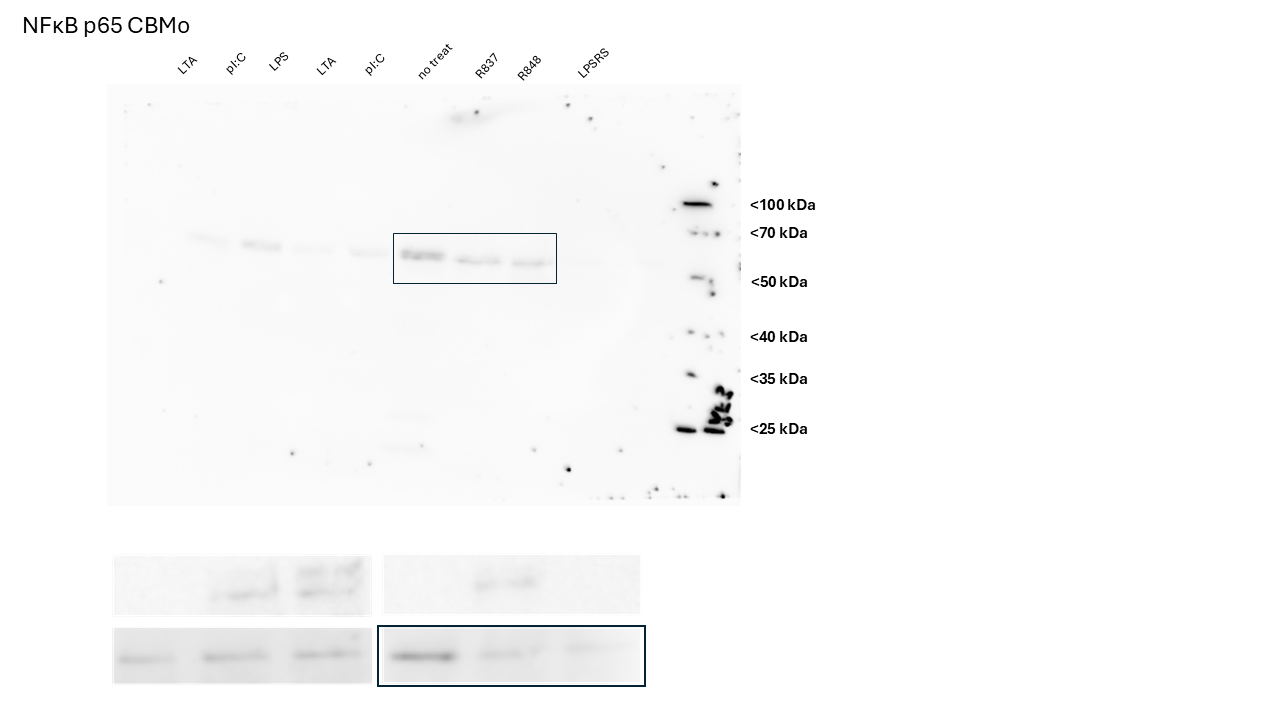

Supplement: Supplementary file 6 — Supplementary Material 6 [file 41598_2026_46534_MOESM6_ESM.png]

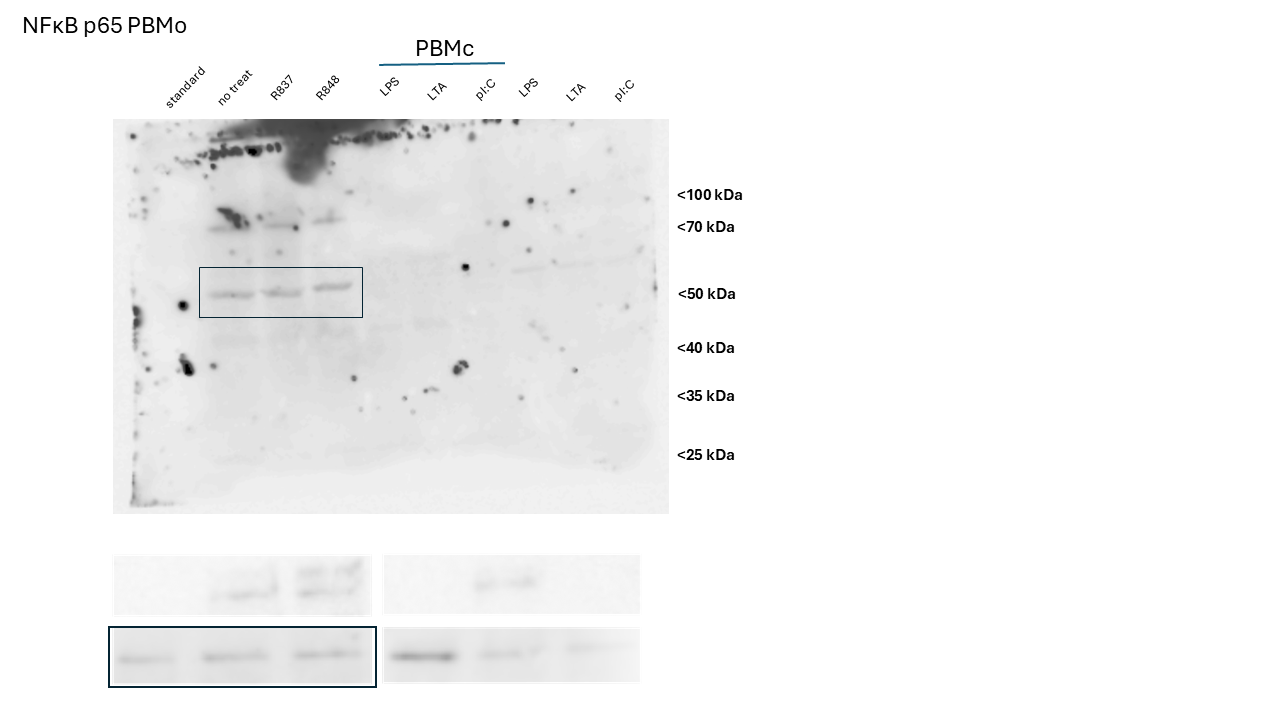

Supplement: Supplementary file 8 — Supplementary Material 8 [file 41598_2026_46534_MOESM8_ESM.png]
